# Pacemaker Pocket Infection After Splenectomy

**DOI:** 10.7759/cureus.35920

**Published:** 2023-03-09

**Authors:** Ramachandra Barik, Pranjit Deb, Abhinav Kumar, Rudrapratap Mahapatra

**Affiliations:** 1 Cardiology, All India Institute of Medical Sciences, Bhubaneswar, IND; 2 Cardiothoracic Surgery, All India Institute of Medical Sciences, Bhubaneswar, IND

**Keywords:** pneumococcal vaccine, reuse of pacemaker, abandoned leads, pacemaker pocket infection, splenectomy

## Abstract

A post-splenectomy patient suffers from frequent infections due to capsulated bacteria like Streptococcus pneumoniae, Hemophilus influenzae, and Neisseria meningitidis despite vaccination because of a lack of memory B lymphocytes. Pacemaker implantation after splenectomy is less common. Our patient underwent splenectomy for splenic rupture after a road traffic accident. He developed a complete heart block after seven years, during which a dual-chamber pacemaker was implanted. However, he was operated on seven times to treat the complication related to that pacemaker over a period of one year because of various reasons, which have been shared in this case report. The clinical translation of this interesting observation is that, though the pacemaker implantation procedure is a well-established procedure, the procedural outcome is influenced by patient factors like the absence of a spleen, procedural factors like septic measures, and device factors like the reuse of an already-used pacemaker or leads.

## Introduction

Pacemaker pocket infection or infective endocarditis due to vegetation over the pacemaker leads has several causes, like patient factors, procedural technique, and the type of pacemaker and its leads. When it occurs, it is associated with high treatment costs, prolonged hospital stays, frequent outpatient visits, morbidity, and in a few cases, may even result in death [1], which adds to the cost [2]. Asplenia or post-splenectomy status causes significant bloodstream infection by capsulated bacteria due to the absence of memory B cells [3]. Pacemaker pocket infection, especially after splenectomy, is less common. We came across a patient who had undergone a splenectomy for a spleen rupture in a road traffic accident in 2014. This patient underwent dual chamber pacemaker implantation after seven years to treat complete heart failure due to sick sinus syndrome. However, the pacemaker pocket had repeated infections, most probably due to impaired immunity because of its post-splenectomy status. The case report is very interesting because the patient was operated on seven times over a period of one year for a pacemaker pocket infection.

## Case presentation

A 56-year-old man presented with a permanent pacemaker pocket infection in the right pectoral area for a month. The detailed case history with respect to the timeline is mentioned in Table 1.

**Table 1:** Timeline of the case PPI: permanent pacemaker implantation; DDD: dual chamber pacemaker; SJ Medical: St. Jude Medical; AIIMS: All India Institute of Medical Sciences

Time	Place/hospital	Event/Procedure	Additional history
2014	Tata Main Hospital, Jamshedpur	Splenectomy for splenic rupture after a road traffic accident.	The pneumococcal vaccine was given twice after the splenectomy.
30/12/2021	Brahmananda Narayana Multispeciality Hospital, Jamshedpur	PPI (DDD, SJ Medical) from the left pectoral area.	Complete heart block.
14/01/2022	Brahmananda Narayana Multispeciality Hospital, Jamshedpur	The pacemaker pocket in the left pectoral area developed signs of infection.	Organism not known.
23/02/2022	Meditrina Super Speciality Hospitals, Jamshedpur	The pacemaker and one pacemaker lead could be explanted, but the pectoral pocket was discharging pus.	This may be due to abandoned lead without capping the cut end in the pacemaker pocket.
01/03/2022	Meditrina Super Speciality Hospitals, Jamshedpur	PPI on the right pectoral area (DDD, SJ medical).	The pacemaker implantation was performed on the right pectoral area to treat the complete heart block even though the pocket on the left pectoral had a chronic infection.
08/07/2022	Meditrina Super Speciality Hospitals. Jamshedpur	The second attempt to remove the abandoned lead failed, and the left side pocket continued to discharge pus. The patient was given several oral or injectable antibiotics, empirically.	
10/10/2022	AIIMS, Bhubaneswar	Right pocket infection reported.	The patient was prescribed oral antibiotics empirically as he was not ready for admission during the same visit.
21/01/2023	AIIMS, Bhubaneswar	Right side pacemaker explanted with both leads.	Leads were screwing type, and straight stylet support was taken to unscrew.
28/01/2023	AIIMS, Bhubaneswar	The left side abandoned lead was explanted with pocket debridement with support from the cardiac surgeon. Electric cautery was used for hemostasis.	A left antecubital venogram revealed a left subclavian vein occlusion.
02/02/203	AIIMS, Bhubaneswar	PPI (DDD, SJ Medical performed) from the right pectoral area near the previous scar. The right subclavian vein was partially occluded.	
13/02/2023	AIIMS, Bhubaneswar	The patient was discharged successfully.	By this time, the patient had been operated on seven times over a period of one year.

**Figure 1:**
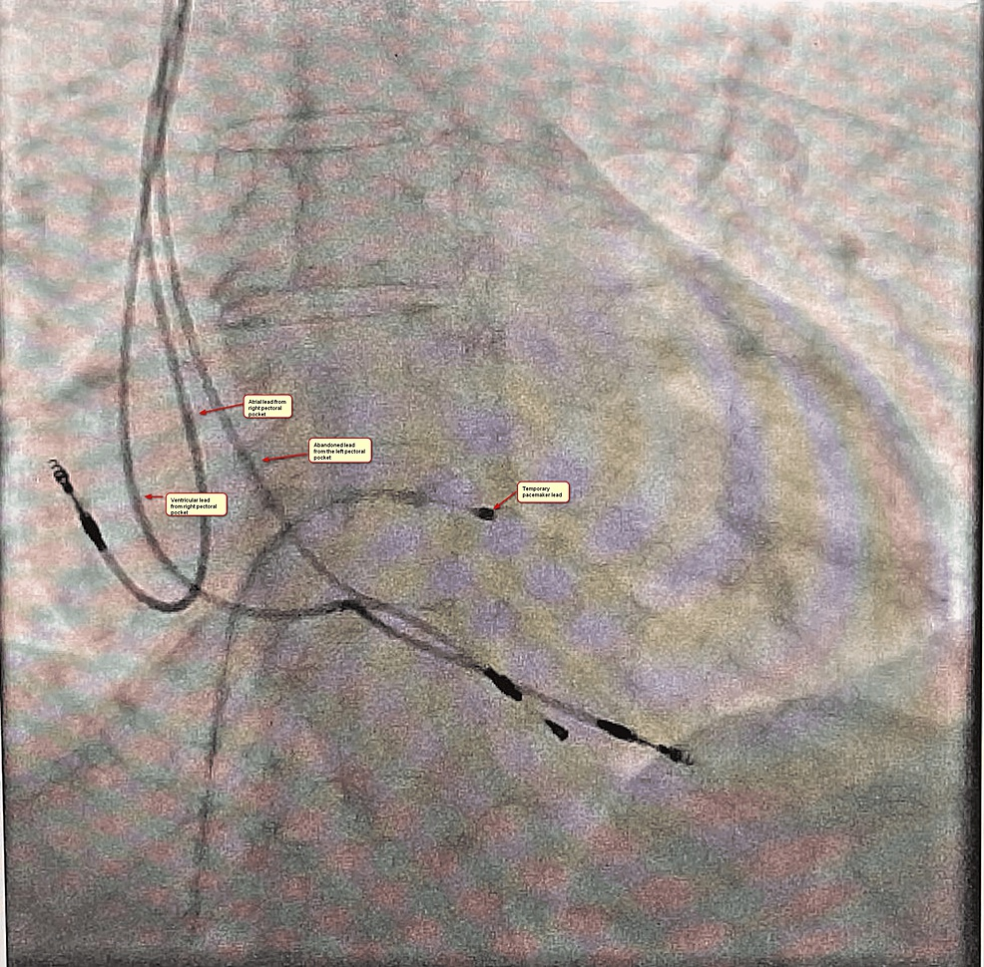
Chest x-ray Chest x-ray (posterior-anterior (PA) view) shows an abandoned left-side lead and two leads from the right-sided infected pacemaker pocket.

History revealed that he had a road traffic accident nine years ago when he sustained a splenic rupture, for which an exploratory laparotomy with splenectomy was done. After a period of seven years after the splenectomy, he developed a complete heart block, for which a permanent pacemaker (dual chamber pacemaker (DDD), St. Jude Medical (SJ Medical)) was implanted in the left pectoral fossa on December 31, 2021. On January 14, 2022, a pacemaker pocket infection was diagnosed, for which the pulse generator and atrial lead were explanted, but the ventricular lead could not be explanted, and the pocket was closed. On March 1, 2022, the sterilised left-sided pacemaker with two new leads (DDD and SJ medical) was implanted in the right pectoral region. In view of reinfection and continuous pus discharge from the left-sided pacemaker site, it was re-explored but failed to be extracted. On October 20, 2022, he reported to us with a right-sided pacemaker pocket infection but refused to get admitted on that visit. He was given oral levofloxacin in combination with ampicillin and sulbactam, and it was suggested that he visit again at the earliest possible time for the proper treatment of the pacemaker pocket infection. On January 20, 2023, he was admitted with a frank pus-discharging pacemaker pocket on the right side, a pulse generator protruding from the old skin incision site, and a discharging sinus tract formation on the left-sided wound. At the time of admission, he was fully conscious, cooperative, coherent, and afebrile, with a heart rate of 77 beats per minute. His blood pressure was 132/80 mm Hg, oxygen saturation (SpO2) was 98% in room air, there was no lymphadenopathy, and his left-sided pacemaker pocket was tender, swollen, and erythematous, suggesting cellulitis. His other systemic physical examination was grossly within normal limits except for a midline scar in the abdomen, suggestive of a previous history of splenectomy. A complete hemogram and liver and kidney function tests were grossly within the normal range. A chest x-ray of a normal lung field with the right-side pectoral pocket pacemaker and two leads connected to it and one left pectoral abandoned ventricular lead was taken (Figure 1).

**Figure 2:**
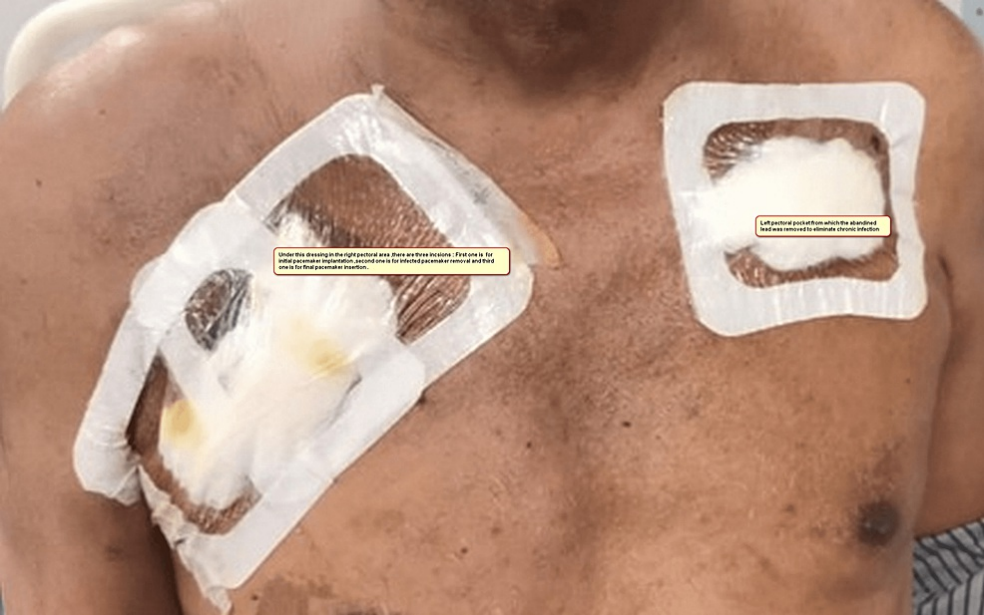
Anterior chest wall The anterior chest wall shows two healed pacemaker implantation sites on the right side and one healed pacemaker site on the left side.

Echocardiography showed good biventricular function and no signs of infective endocarditis. With temporary pacemaker support through the right femoral approach, a right-sided pacemaker, and both the leads and the pocket closed, keeping the drain in situ. On January 28, 2023, while he was on temporary pacemaker support under general anaesthesia, the left-sided pocket was re-explored with the support of a cardiothoracic and vascular (CTVS) surgeon, and abandoned lead extraction and wound debridement were performed. A drain was left in place. We used intravenous tranexamic acid throughout the procedures. His left antecubital venogram showed left subclavian vein occlusion. The pus samples that were sent from both infected wounds were sterile. As the patient requested a DDD pacemaker only and not epicardial pacing, on February 2, 2023, a permanent pacemaker (DDD, SJ Medical) was implanted in the right pre-pectoral area, near the previous scar. After 21 days of hospitalisation, the patient was successfully discharged, giving us a unique experience (Figure 2). The microscopic examination of urine showed plenty of pus cells, and the culture of urine yielded E. coli, which was resistant to most of the routinely used antibiotics except amikacin.

## Discussion

Pacemaker pocket infection is defined as the presence of local signs of inflammation at the site of the pacemaker pocket [4]. It may be associated with vegetation over the implanted pacemaker leads and heart valves, pulmonary thromboembolism, and other systemic infections due to bacteremia. By the time a patient goes to another hospital for proper care, the pacemaker is usually out of pocket, as in our case. The rate of pocket infection is significantly higher after reimplantation procedures [5]. Nearly 15% of the patients with pacemaker pocket infections may have systemic infections.

Risk factors for pacemaker pocket infection can be divided into three categories: patient-related, procedure-related, and device-related. Some of these are modifiable factors. Identification of modifiable risk factors is important because that may help to take preventive measures to reduce the risk of recurrent infections. Our patient had three apparent risk factors, namely post-splenectomy status, an abandoned pacemaker lead in the left pectoral pocket, and the reuse of a sterilised pacemaker (Table 1). Recurrent infection of the pacemaker pocket, as in our case, has not been reported earlier. It may be due to the repeated use of several empiric antibiotic regimens one after another that our patient did not grow bacteria on a blood or pus culture from the infected pocket, or the organism may be atypical. Our patient received the pneumococcal vaccination twice. In our case, the infection related to the abandoned lead on the left and the reuse of a sterilised pacemaker could have been prevented to reduce recurring infection [6,7].

Chronic kidney disease, diabetes mellitus, chronic obstructive pulmonary disease, corticosteroid use, a history of previous device infection, malignancy, heart failure, pre-procedural infection in the body, anticoagulant or antiplatelet use, and skin disorders are important patient-related risk factors. The procedure-related factors are lack of proper hand washing, an inadequate infection control programme in the hospital, no standardised sterilisation protocol, pocket hematoma, poor hemostasis due to the lack of all the layers being stitched systematically, early lead repositioning, and inexperienced operators [8]. The device-related factors are few but have a significant impact on the incidence rate of pacemaker pocket infections. The abdominal pocket site, a heavier device, a greater number of leads (including epicardial leads), and the reuse of sterile pacemakers or leads significantly increase pacemaker pocket infection and infective endocarditis, as in our case [4,9]. Cultures from pocket sites, lead tips, and blood samples may become positive in 60 to 70% of the patients, but these were negative in our case because of repeated use of empirical antibiotics or because the patient had an atypical bacterial infection [10]. As our patient had undergone splenectomy earlier, the multiple blood cultures or pus from both the pacemaker site were sent for aerobic, anaerobic, and fungal cultures, but none had growth of any organism. The reasons for repeated pacemaker infections in our case may be related to the post-splenectomy status, the re-use of an explanted pacemaker, abandoned leads without capping the cut end of them in the pacemaker packet, and the treatment of the patient in multiple centres. However, we feel the post-splenectomy status is the main reason for repeated infections. Even though there is no evidence of encapsulated bacterial growth in the blood or pus culture in our case, the repeated use of antibiotics can be explained. As we notice in everyday practice, all the risk factors except the splenectomy are usual risk factors, and they do not pose great challenges, i.e., seven operations by different experts over a period of one year.

## Conclusions

Multiple factors that can lead to recurrent pacemaker pocket infections are patient-related, device-related, or procedure-related. A recurring pacemaker pocket infection in a post-splenectomy patient has not been reported earlier in this report. The other risk factors for recurrent pacemaker pocket infection in our case are things like abandoned leads on the left side, the reuse of a sterile pacemaker, and referrals for pocket infection to other centres. The latter risk factors usually do not pose great challenges, as in our case, where the patient needed seven surgeries over a period of one year, which makes this pacemaker pocket infection so interesting. The absence of bacterial growth in our case can be explained by the previous pneumococcal vaccination and partial treatment with several regimens of empirical antibiotics.
